# The Clinical Significance of the Morning Blood Pressure Surge in Chronic Kidney Disease: A Systematic Review

**DOI:** 10.7759/cureus.103347

**Published:** 2026-02-10

**Authors:** Hina Faiz, Ahmad Mohammad, Abdulaziz Tageldin Ahmed Mahmoud, Isaac J Parada Buenaventura, Leen I Sabbagh, Jasreen Singh, Asad Raza

**Affiliations:** 1 Internal Medicine, Combined Military Hospital, Rawalakot, PAK; 2 Internal Medicine, Hurley Medical Center, Flint, USA; 3 Internal Medicine, Medical University of Lodz, Lodz, POL; 4 Cardiology, Hospital Miguel H. Alcívar, Bahía de Caraquez, ECU; 5 Obstetrics and Gynecology, Dubai Medical College for Girls, Dubai, ARE; 6 Internal Medicine, St. George's University, True Blue, GRD; 7 Internal Medicine, Lahore Medical and Dental College, Lahore, PAK

**Keywords:** ambulatory blood pressure monitoring, chronic kidney disease, circadian blood pressure, morning blood pressure surge, renal outcomes

## Abstract

Morning blood pressure surge (MBPS) is a circadian blood pressure phenomenon implicated in cardiovascular and renal risk. Patients with chronic kidney disease (CKD) frequently exhibit circadian BP disturbances, but the prognostic significance of MBPS in this population remains uncertain. This systematic review evaluated the association between MBPS and clinical outcomes in adults with established CKD. A comprehensive search of major electronic databases identified observational cohort studies assessing MBPS using 24-hour ambulatory blood pressure monitoring. Studies reporting cardiovascular events, renal outcomes, or mortality were included. Two cohort studies comprising 457 patients met eligibility criteria. In one study, elevated MBPS (≥35 mmHg) was independently associated with a higher risk of composite adverse outcomes (HR 3.12; 95% CI 1.10-9.13). In the second study, elevated MBPS predicted CKD progression (HR 2.35; 95% CI 1.20-4.63) but showed no significant association with cardiovascular events or mortality. Despite differences in MBPS definitions and outcome measures, both studies demonstrated that MBPS provides independent prognostic information. Current evidence, though limited, indicates that elevated MBPS is associated with adverse renal and composite clinical outcomes in CKD. These findings support the potential utility of ambulatory BP monitoring and circadian BP assessment in risk stratification. Larger prospective studies are needed to clarify cardiovascular implications and determine whether targeting MBPS can improve outcomes.

## Introduction and background

Chronic kidney disease (CKD) is a major global public health problem and is associated with a markedly increased risk of cardiovascular morbidity and mortality. Cardiovascular disease is the leading cause of death among patients with CKD across all stages of renal impairment [1,2]. Hypertension is highly prevalent in this population and plays a central role in the development and progression of both cardiovascular and renal complications. However, conventional clinic blood pressure measurements often fail to capture the full cardiovascular risk profile of patients with CKD [3,4].

Hypertension in CKD is characterized by complex pathophysiological mechanisms, including volume overload, sympathetic nervous system activation, renin-angiotensin-aldosterone system dysregulation, endothelial dysfunction, and increased arterial stiffness [5,6]. These abnormalities contribute to disturbed circadian blood pressure regulation. Ambulatory blood pressure monitoring has shown that patients with CKD frequently exhibit non-dipping patterns, nocturnal hypertension, and increased blood pressure variability. These circadian abnormalities are independently associated with adverse cardiovascular and renal outcomes and provide prognostic information beyond office blood pressure measurements [7].

Compared with individuals who have essential hypertension, patients with CKD demonstrate more profound disruptions in circadian blood pressure behavior. While essential hypertension typically retains a relatively preserved nocturnal dipping pattern and a physiologic early-morning rise, CKD is associated with a markedly higher prevalence of nocturnal hypertension, reverse dipping, blunted or inconsistent sleep-wake transitions, and greater sensitivity to sympathetic and sodium-mediated fluctuations. These distinctions may magnify the hemodynamic burden imposed by early-morning blood pressure changes.

Morning blood pressure surge (MBPS) refers to the rapid rise in blood pressure occurring during the transition from sleep to wakefulness. Two commonly used definitions are cited in the literature: the sleep-trough morning surge, calculated as the difference between morning systolic blood pressure and the lowest nocturnal systolic pressure, and the pre-waking surge, defined as the difference between morning systolic blood pressure and the average systolic pressure during the two hours preceding awakening. This physiologic rise is mediated by heightened sympathetic activation, hormonal shifts, and increased vascular tone. An exaggerated morning surge has been associated with endothelial injury, vascular inflammation, and plaque instability [8], and in the general population, excessive MBPS has been linked to higher risks of stroke, acute coronary syndromes, and cardiovascular mortality [9].

Patients with CKD may be particularly vulnerable to the adverse effects of an elevated MBPS. Autonomic dysfunction, impaired sodium handling, and increased arterial stiffness can amplify these fluctuations. Additionally, CKD is characterized by heightened systemic inflammation and vascular calcification, both of which may increase susceptibility to surge-related vascular injury [10]. Although emerging studies have examined the relationship between MBPS and outcomes in CKD, the evidence remains limited, and the prognostic significance of this parameter has not been comprehensively synthesized.

The objective of this systematic review is to evaluate the prognostic value of MBPS in adult patients with CKD. This review specifically examines the association between MBPS, as measured by ambulatory blood pressure monitoring, and both cardiovascular and renal outcomes, providing a balanced assessment of its relevance across these two critical clinical domains.

## Review

Materials and methods

Study Design and Reporting Standards

This systematic review was conducted to evaluate the prognostic value of MBPS in patients with CKD. The review was designed and reported in accordance with the Preferred Reporting Items for Systematic reviews and Meta-Analyses (PRISMA) guidelines [11]. A structured and transparent methodology was applied to ensure reproducibility and minimize bias throughout the study selection and data synthesis processes.

Research Question and Population, Exposure, Comparator, Outcome (PICO) Framework

The research question was formulated using the PICO framework [12]. The population of interest included adult patients aged 18 years or older with a confirmed diagnosis of CKD who were not receiving dialysis at baseline. The exposure of interest was the elevated MBPS, as assessed using 24-hour ambulatory blood pressure monitoring. The comparator consisted of patients with CKD who did not demonstrate an elevated MBPS, as defined by study-specific thresholds. The outcomes of interest included cardiovascular events, CKD progression, end-stage renal disease, and all-cause mortality. This framework guided the development of the search strategy, eligibility criteria, and data extraction process. Although all included studies assessed MBPS using established ambulatory blood pressure monitoring-based definitions, the specific operationalization and threshold used to define an “elevated” surge were study-dependent rather than fully standardized.

Literature Search Strategy

A comprehensive literature search was conducted across multiple electronic databases, including PubMed, Embase, and the Cochrane Library, to identify relevant studies from database inception through the most recent search date. The search strategy combined controlled vocabulary terms and free-text keywords related to MBPS and CKD, using Boolean operators. The core search terms included combinations of “morning blood pressure surge” OR “morning blood pressure increase” OR “morning BP surge” AND “chronic kidney disease” OR “CKD” OR “chronic renal insufficiency”, along with prognostic and outcome-related terms such as “prognosis”, “outcomes”, “cardiovascular events”, “renal progression”, and “mortality”. Searches were restricted to studies involving human participants and published in the English language. In addition, the reference lists of all included articles were manually screened to identify any relevant studies that may not have been retrieved through the electronic database search.

Study Selection and Eligibility Criteria

Studies were screened in two stages. Titles and abstracts were initially reviewed to exclude clearly irrelevant articles. A full-text review was subsequently performed to assess eligibility based on predefined inclusion and exclusion criteria. Studies were included if they enrolled adult patients with established CKD, evaluated MBPS using ambulatory blood pressure monitoring, and reported associations with cardiovascular or renal outcomes. Observational cohort studies were eligible for inclusion. Studies were excluded if they focused on incident CKD in populations without baseline renal disease, did not assess MBPS, relied solely on office or home blood pressure measurements, or lacked relevant outcome data. Disagreements regarding study eligibility were resolved through discussion and consensus.

Data Extraction

Data extraction was performed using a standardized data collection table developed specifically for this review. Extracted variables included study identifiers, sample size, characteristics of the CKD population, ambulatory blood pressure monitoring methodology, definition and categorization of MBPS, duration of follow-up, outcomes assessed, and key adjusted effect estimates. Data were extracted directly from full-text articles to ensure accuracy and completeness. The extracted information was cross-checked to minimize errors and inconsistencies.

Risk of Bias Assessment

The methodological quality and risk of bias of the included studies were assessed using the Newcastle-Ottawa Scale for cohort studies [13]. This tool evaluates bias across three domains, including selection of study cohorts, comparability of exposed and nonexposed groups, and outcome assessment. Each study was independently evaluated, and the overall risk of bias was categorized based on total scores. The results of the risk of bias assessment are presented descriptively and summarized in a dedicated table.

Data Synthesis

Given the limited number of eligible studies and the heterogeneity in outcome definitions and follow-up durations, a quantitative meta-analysis was not performed. Instead, a qualitative narrative synthesis was conducted. Study findings were compared and contrasted with respect to population characteristics, exposure definitions, outcome measures, and adjusted prognostic associations. Particular attention was given to differences between cardiovascular and renal outcome findings. The synthesis focused on identifying consistent patterns, potential sources of heterogeneity, and clinically relevant insights.

Ethical Considerations

As this study was a systematic review of previously published literature, ethical approval and informed consent were not required. All included studies had received appropriate ethical approval as reported by their respective authors.

Results

Study Selection Process

**Figure 1:**
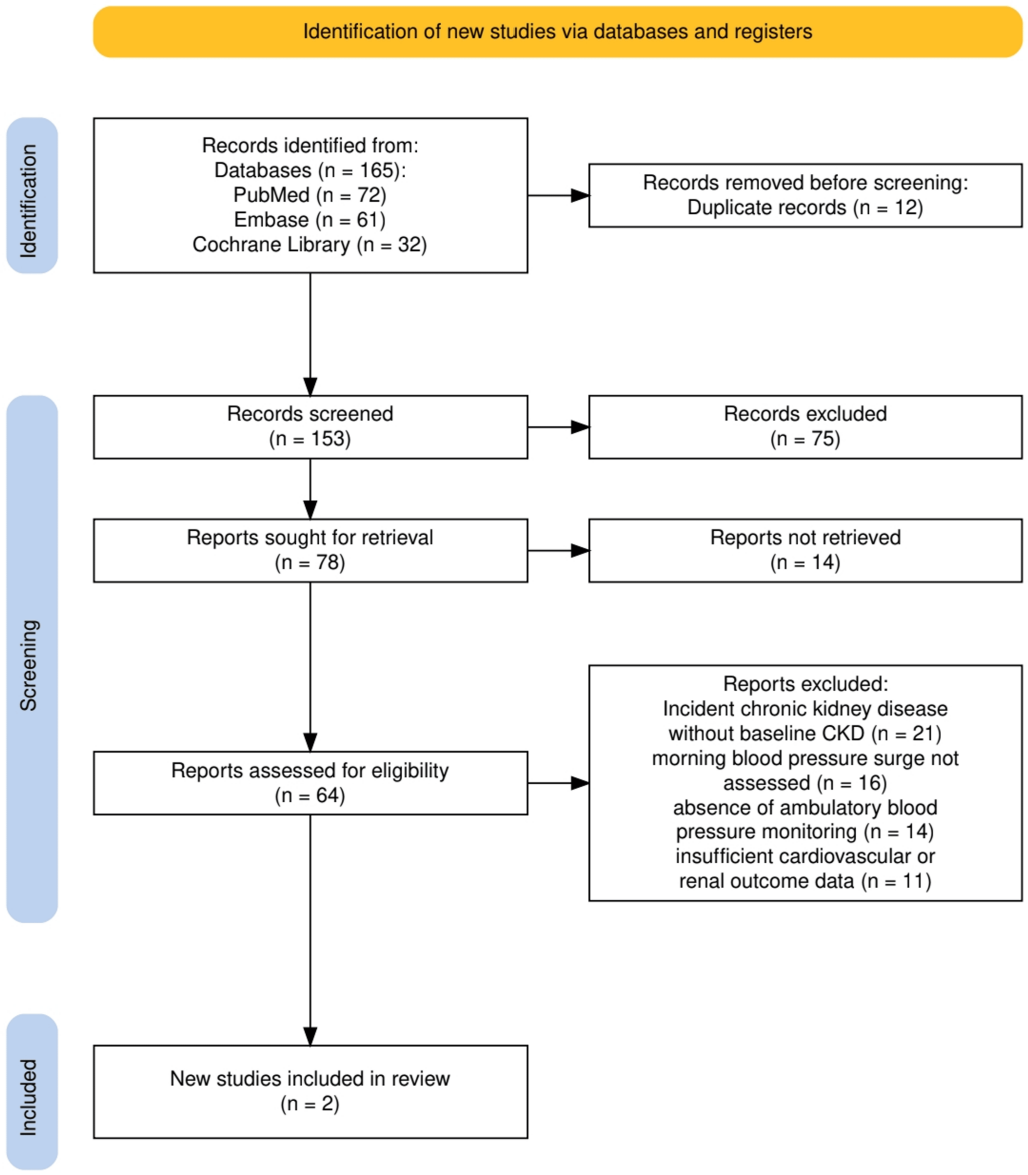
PRISMA flow diagram showing the study selection process CKD, chronic kidney disease; PRISMA, Preferred Reporting Items for Systematic reviews and Meta-Analyses

The study selection process is illustrated in Figure 1. A total of 165 records were identified through electronic database searching, including PubMed, Embase, and the Cochrane Library. After removal of duplicate records, 153 unique records were screened based on titles and abstracts, of which 75 were excluded for lack of relevance. Seventy-eight reports were subsequently sought for full-text retrieval, and 14 reports could not be retrieved. Sixty-four full-text articles were assessed for eligibility. Of these, 62 studies were excluded due to predefined criteria, including a focus on incident CKD without baseline renal disease, absence of MBPS assessment, lack of ambulatory blood pressure monitoring, or insufficient cardiovascular or renal outcome data. Ultimately, two observational studies met the inclusion criteria and were included in the qualitative synthesis of this systematic review.

Characteristics of the Selected Studies

The key characteristics of the included studies are summarized in Table 1. A total of two observational cohort studies were included, encompassing 457 adult patients with CKD. Both studies enrolled nondialysis patients and evaluated MBPS using 24-hour ambulatory blood pressure monitoring. Sample sizes ranged from 153 to 304 participants, with follow-up durations varying from a median of 30 months to a mean of 4.3 years. MBPS was defined consistently across studies as the difference between morning systolic blood pressure and the lowest nocturnal systolic blood pressure, with patients categorized according to predefined surge thresholds. The primary outcomes assessed included composite cardiovascular and renal events, CKD progression, and all-cause mortality. Both studies employed multivariable Cox proportional hazards models to evaluate the association between elevated MBPS and clinical outcomes, adjusting for relevant demographic, clinical, and blood pressure-related confounders. Despite differences in study populations and outcome definitions, the methodological approaches were largely comparable, allowing for a structured qualitative synthesis of findings.

**Table 1:** Summary of included studies evaluating MBPS in CKD ABPM, ambulatory blood pressure monitoring; BP, blood pressure; CKD, chronic kidney disease; ESRD, end-stage renal disease; MBPS, morning blood pressure surge

Study	Sample size (n)	CKD population	Mean age (years)	Male (%)	Hypertension (%)	ABPM method	Definition of morning BP surge	MBPS cutoff/categories	Follow-up duration	Outcome	Key findings (adjusted results)
Ma et al. (2024) [14]	153	Adult CKD patients (≥18 years), nondialysis	41.8	56.9	Not explicitly reported	24-hour ambulatory BP monitoring	Difference between morning systolic BP and lowest nighttime systolic BP	MBPS ≥35 mmHg vs. <35 mmHg	Mean 4.3 years (minimum 1 year)	Composite endpoint of cardiovascular events, cerebrovascular events, end-stage renal disease, and all-cause mortality	MBPS was independently associated with a higher risk of composite outcomes (HR 3.124, 95% CI 1.096-9.130). Systolic BP and daytime and nighttime pulse pressures were also independently associated with outcomes
Liu et al. (2021) [15]	304	Adult patients with CKD and hypertension, nondialysis	Not explicitly reported	Not explicitly reported	100	24-hour ambulatory BP monitoring	Difference between morning systolic BP and lowest nighttime systolic BP	Elevated MBPS vs. non-elevated MBPS (cutoff defined by study)	Median 30 months	CKD progression, cardiovascular events, and all-cause mortality	Elevated MBPS independently predicted CKD progression (HR 2.35, 95% CI 1.20-4.63, p = 0.013), independent of morning BP. No significant association was observed between elevated MBPS and cardiovascular events (HR 1.02, 95% CI 0.66-1.57) or all-cause mortality (HR 1.08, 95% CI 0.46-2.55)

Quality Assessment

The risk of bias of the included studies was assessed using the Newcastle-Ottawa Scale for cohort studies, as summarized in Table 2. Overall, both studies were judged to have a low risk of bias across the key methodological domains. In the selection domain, the cohorts were representative of adult patients with CKD, and MBPS was clearly defined and measured using standardized 24-hour ambulatory blood pressure monitoring. Regarding comparability, both studies employed multivariable regression models and adjusted for major potential confounders, including demographic factors, baseline blood pressure parameters, and renal function indices. Outcome assessment was robust in both studies, with clearly defined cardiovascular and renal endpoints and adequate follow-up duration to capture clinically relevant events. Minor limitations were noted, primarily related to the observational nature of the studies and, in one study, the retrospective design. However, these factors were not considered sufficient to substantially compromise internal validity. Collectively, the methodological quality of the included studies supports the reliability of the reported associations between MBPS and adverse outcomes in patients with CKD.

**Table 2:** Risk of bias assessment of studies evaluating MBPS in CKD CKD, chronic kidney disease; MBPS, morning blood pressure surge

Study	Selection (0-4)	Comparability (0-2)	Outcome (0-3)	Total score (0-9)	Overall risk of bias
Ma et al. (2024) [14]	3	2	3	8	Low risk
Liu et al. (2021) [15]	4	2	3	9	Low risk

Discussion

This systematic review synthesizes the limited available evidence examining the prognostic relevance of MBPS in patients with CKD. Across the two included studies, an elevated MBPS demonstrated an association with adverse clinical outcomes; however, the findings were not fully consistent. One study reported a significant association between elevated morning surge and CKD progression, whereas the other did not identify a clear relationship with renal or cardiovascular endpoints [16]. Given this variability and the very small number of eligible studies, any interpretation regarding a preferential prognostic signal for renal outcomes must be made cautiously. Instead, the current evidence should be viewed as preliminary and hypothesis-generating, highlighting the need for further investigation rather than establishing definitive prognostic patterns. Even so, both studies underscore the potential clinical relevance of circadian blood pressure dynamics in CKD and support continued exploration of MBPS as an underrecognized risk phenotype.

The possible association between MBPS and adverse outcomes in CKD can be understood within established pathophysiological mechanisms. CKD is characterized by heightened sympathetic nervous system activity, impaired sodium excretion, and persistent volume expansion, all of which contribute to abnormal circadian blood pressure regulation [17]. Increased arterial stiffness and reduced vascular compliance, which are highly prevalent in this population, further amplify pulsatile hemodynamic stress during the early morning hours. Additionally, nocturnal hypertension and reduced dipping are common in CKD and may influence the apparent magnitude of the surge. Importantly, even a modest rise in morning blood pressure may exert disproportionate hemodynamic stress on structurally vulnerable renal microvasculature, potentially promoting glomerular hypertension, endothelial injury, and nephron loss [18,19]. These mechanistic pathways provide biological plausibility for the renal findings observed in one of the included studies, while also helping to contextualize the heterogeneity in cardiovascular outcomes reported across the limited evidence base.

The prognostic associations observed for cardiovascular and renal outcomes differed across the included studies and therefore warrant cautious interpretation. In the study by Ma et al. [14], an elevated MBPS was associated with a composite endpoint incorporating cardiovascular events, end-stage renal disease, and mortality. In contrast, the study by Liu et al. reported a significant association between elevated morning surge and CKD progression but did not identify meaningful associations with cardiovascular events or death. Several factors may account for these discrepancies. Both cohorts were relatively small, which limited statistical power, particularly for cardiovascular outcomes that generally occur less frequently and often require longer follow-up to capture. The study populations were also comparatively young, with fewer established cardiovascular comorbidities, further reducing event incidence. Additionally, follow-up duration may have been adequate to observe renal deterioration but insufficient to detect overt cardiovascular manifestations [20]. Variability in outcome definitions, such as the use of composite endpoints versus isolated cardiovascular events, may also have contributed to divergent findings. Residual confounding from nocturnal blood pressure and pulse pressure, both strong predictors of cardiovascular risk in CKD, may further obscure independent associations. Another important consideration is the use and timing of antihypertensive medication, which can substantially influence early morning hemodynamics and therefore modify the magnitude of the observed surge; neither study was able to fully account for this potential confounder. Taken together, these observations suggest that any apparent sensitivity of renal outcomes to circadian blood pressure stress should be interpreted as a preliminary signal rather than a definitive or consistent pattern across studies.

Several methodological challenges inherent to MBPS research limit comparability and complicate interpretation. There is currently no universally accepted definition of MBPS, and cutoff values used to define an “elevated” surge vary across studies, contributing to exposure heterogeneity. Most investigations rely on a single ambulatory blood pressure monitoring session, which may not fully reflect day-to-day variability in circadian blood pressure behavior [21]. Moreover, nocturnal blood pressure and dipping status exert a strong influence on the calculated magnitude of the surge; higher nighttime blood pressure can attenuate the apparent surge while independently predicting adverse outcomes. Medication timing represents an additional source of variability, especially in patients receiving antihypertensive therapy that may alter early morning hemodynamics. These limitations emphasize the importance of interpreting MBPS within the broader context of circadian blood pressure regulation rather than as an isolated or standalone parameter [22].

When considered alongside other blood pressure phenotypes, MBPS occupies a complementary but distinct role in cardiovascular and renal risk assessment. Morning hypertension reflects absolute blood pressure levels after awakening, while nocturnal hypertension and non-dipping patterns capture sustained nighttime hemodynamic load, both of which have well-established prognostic significance in CKD [8]. Blood pressure variability represents another dimension of vascular stress linked to target organ damage. MBPS, by contrast, reflects the dynamic transition from nocturnal to daytime blood pressure and may provide incremental prognostic information related to vascular reactivity and autonomic regulation. When combined with nocturnal blood pressure measurements, assessment of MBPS may enhance identification of high-risk patients who appear adequately controlled on clinic measurements alone [23]. This integrated approach underscores the potential value of comprehensive ambulatory blood pressure phenotyping in improving risk stratification in CKD.

The findings of this systematic review suggest that assessment of MBPS may offer incremental value in cardiovascular and renal risk stratification among patients with CKD. Incorporating ambulatory blood pressure monitoring into routine clinical evaluation may improve detection of circadian blood pressure abnormalities that are not captured by office measurements alone [24]. Identification of elevated MBPS may help clinicians recognize patients who are at higher risk of disease progression and who may benefit from closer monitoring and more individualized blood pressure assessment. However, the current evidence does not support routine therapeutic interventions based solely on MBPS, nor does it allow firm conclusions regarding the clinical benefit of chronotherapy in this population. As such, MBPS should be viewed as a complementary risk marker rather than a standalone treatment target.

Several limitations should be considered when interpreting the findings of this review. At the study level, the number of available investigations was small, and all included studies employed observational designs, which preclude causal inference. Differences in outcome definitions and follow-up duration contributed to heterogeneity across studies. At the review level, the limited number of eligible studies precluded quantitative synthesis, and a meta-analysis was therefore not performed. The restricted evidence base may also limit external validity, particularly across different CKD stages and ethnic populations. In addition, the possibility of publication bias cannot be excluded, as studies reporting null associations may be underrepresented. These limitations highlight important knowledge gaps rather than weaknesses and underscore the need for further research in this emerging area.

Future research should focus on large, well-designed prospective cohort studies specifically enrolling patients with CKD to further clarify the prognostic significance of MBPS. Standardization of MBPS definitions and cutoff values is essential to improve comparability across studies. Repeated ambulatory blood pressure monitoring assessments would allow evaluation of the stability of circadian blood pressure patterns over time and their relationship with long-term outcomes. Integrating MBPS with other circadian blood pressure parameters, including nocturnal hypertension and blood pressure variability, may provide a more comprehensive risk profile. Ultimately, randomized controlled trials are needed to determine whether interventions targeting circadian blood pressure abnormalities can improve cardiovascular and renal outcomes. In this context, MBPS may emerge as a component of personalized blood pressure phenotyping in CKD, informing individualized risk assessment and management strategies.

## Conclusions

This systematic review synthesizes the very limited evidence available on the prognostic relevance of MBPS in CKD, drawing on only two observational studies that used ambulatory blood pressure monitoring. Although the findings were not fully consistent, both studies suggest that MBPS may have clinical relevance within the broader context of circadian blood pressure abnormalities in this population. Given the small evidence base, heterogeneity in outcome definitions, and methodological limitations, these observations should be considered preliminary rather than definitive. Nonetheless, the review highlights an important area of emerging interest and underscores the need for larger, well-designed studies that employ standardized definitions, account for confounding factors such as nocturnal blood pressure and antihypertensive medication timing, and clarify whether MBPS offers incremental value in predicting renal or cardiovascular outcomes among patients with CKD.
